# Diabetes treatment intensification and associated changes in HbA1c and body mass index: a cohort study

**DOI:** 10.1186/s12902-016-0101-2

**Published:** 2016-06-02

**Authors:** Christianne L. Roumie, Robert A. Greevy, Carlos G. Grijalva, Adriana M. Hung, Xulei Liu, Marie R. Griffin

**Affiliations:** Veterans Health Administration-Tennessee Valley Healthcare System Geriatric Research Education Clinical Center (GRECC), HSR&D Center, Nashville, TN USA; Department of Medicine, Vanderbilt University, Nashville, TN USA; Department of Health Policy, Vanderbilt University, Nashville, TN USA; Department of Biostatistics, Vanderbilt University, Nashville, TN USA; Nashville VA Medical Center, 1310 24th Ave South GRECC, Nashville, TN 37212 USA

**Keywords:** Diabetes mellitus type 2, Patterns of care, Clinical effectiveness, treatment intensification, Adherence, Clinical outcomes

## Abstract

**Background:**

To describe common type 2 diabetes treatment intensification regimens, patients’ characteristics and changes in glycated hemoglobin (HbA1c) and body mass index (BMI).

**Methods:**

We constructed a national retrospective cohort of veterans initially treated for diabetes with either metformin or sulfonylurea from 2001 through 2008, using Veterans Health Administration (VHA) and Medicare data. Patients were followed through September, 2011 to identify common diabetes treatment intensification regimens. We evaluated changes in HbA1c and BMI post-intensification for metformin-based regimens.

**Results:**

We identified 323,857 veterans who initiated diabetes treatment. Of these, 55 % initiated metformin, 43 % sulfonylurea and 2 % other regimens. Fifty percent (*N* = 89,057) of metformin initiators remained on metformin monotherapy over a median follow-up 58 months (interquartile range [IQR] 35, 74). Among 80,725 patients who intensified metformin monotherapy, the four most common regimens were addition of sulfonylurea (79 %), thiazolidinedione [TZD] (6 %), or insulin (8 %), and switch to insulin monotherapy (2 %). Across these regimens, median HbA1c values declined from a range of 7.0–7.8 % (53–62 mmol/mol) at intensification to 6.6–7.0 % (49–53 mmol/mol) at 1 year, and remained stable up to 3 years afterwards. Median BMI ranged between 30.5 and 32 kg/m^2^ at intensification and increased very modestly in those who intensified with oral regimens, but 1–2 kg/m^2^ over 3 years among those who intensified with insulin-based regimens.

**Conclusions:**

By 1 year post-intensification of metformin monotherapy, HbA1c declined in all four common intensification regimens, and remained close to 7 % in subsequent follow-up. BMI increased substantially for those on insulin-based regimens.

**Electronic supplementary material:**

The online version of this article (doi:10.1186/s12902-016-0101-2) contains supplementary material, which is available to authorized users.

## Background

Metformin, a biguanide approved and marketed in the U.S. since 1995, is the preferred oral hypoglycemic medication for initial management of type 2 diabetes [1–4]. In 2006, after review of available evidence, the consensus statement by the American Diabetes Association and the European Association for the Study of Diabetes recommended lifestyle modification and metformin as the preferred first line therapies for type 2 diabetes, with a treatment goal being glycated hemoglobin (HbA1C) of ≤7 %. This recommendation was based on evidence from the United Kingdom Prospective Diabetes Study which demonstrated that those randomized to metformin experienced 42 % fewer diabetes-related deaths and 36 % fewer all-cause deaths compared to the diet alone arm [5, 6].

Despite consensus on initial diabetes treatment, when to intensify and the preferred specific treatment regimen remains highly patient specific. Diabetes treatment guidelines have advocated for a shared approach between provider and patient when choosing both the treatment goals and regimen for intensification [7]. A number of factors influence providers to recommend a medication for intensification including medication costs, side effects, their own knowledge and beliefs about future treatment benefits, such as prevention or delay of macrovascular and microvascular disease [8]. Patients’ understanding of their illness, adherence to their treatment regimens, and ability to manage medications also affect decision-making about treatment choices [9, 10]. Finally, health system factors including achievement of performance metrics can drive the decision to intensify therapy [11, 12]. Although many experts recommend titration of therapy to reach HbA1c goals of <7 %, VHA uses an algorithm which determines an individual glycemic target based on consideration of microvascular complications and co morbid illness (Table 1) [13]. The guidelines note multiple treatment options as acceptable add-on medications. Oral medications in general are easier to initiate, but insulin dose can be modified in response to daily variation in food intake, exercise or other variables that cause daily fluctuation in glucose values. Because of the potential microvascular benefit to patients with tighter glycemic control, there has been a fairly substantial increase in the use of insulin over time [14].

**Table 1:** Veterans Health Administration and Department of Defense Clinical Practice Guideline for the management of Diabetes--Glycemic targets [13]

Comorbidity^a^ or Physiologic age	Microvascular complications^b^		
	Absent/Mild	Moderate	Advanced
Absent	<7 %	<8 %	8–9 %
Life expectancy >10 years	(<53 mmol/mol)	(<64 mmol/mol)	(64–75 mmol/mol)
Comorbidity present	<8 %	<8 %	8–9 %
Life expectancy 5–10 years	(<64 mmol/mol)	(<64 mmol/mol)	(64–75 mmol/mol)
Multi Comorbidities	8–9 %	8–9 %	8–9 %
Life expectancy <5 years	(64–75 mmol/mol)	(64–75 mmol/mol)	(64–75 mmol/mol)

^a^Comorbidity includes, but is not limited to, any or several of the following conditions: cardiovascular disease, chronic kidney disease, chronic obstructive pulmonary disease, liver disease, stroke, and malignancy

^b^Microvascular disease includes complications of diabetes: retinopathy, nephropathy (micro or macroalbuminuria) and neuropathy

Given the varied options available for add-on treatment after metformin, and the use of differing HbA1c goals for intensification, variation in clinical practice is likely to result. Little is known about the current clinical practice patterns for patients who fail initial diabetes treatment. Given the recent increase in early insulin use our aim was to describe common treatment intensification patterns after metformin monotherapy, the characteristics of patients prescribed specific regimens and changes in glycated hemoglobin (HbA1c) and body mass index (BMI) post-intensification.

## Methods

### Study design and data sources

We assembled a retrospective cohort of patients seen in Veterans Health Administration (VHA) facilities who had a new hypoglycemic prescription between October 1, 2001 and September 30, 2008 [15, 16]. Patients were considered new-users of a hypoglycemic medication if they had evidence of VHA healthcare utilization in the previous year, and had not filled any oral or injectable diabetic drug within the past 180 days according to a new user design [17].

VHA pharmacy data identified dispensed prescriptions, including medication name, date filled, days supplied, pill or vial number and dosage [18]. VHA medical datasets containing demographic data and ICD9-CM coded diagnostic and procedure information identified inpatient and outpatient encounters [19]. Laboratory test results were collected from standard VHA clinical sources. Vital signs data included all outpatient measurements of height, weight and blood pressure. For Medicare or Medicaid enrollees, we obtained supplemental encounter, prescription (Part D) and race data from the Centers for Medicare and Medicaid Services through VHAs interagency exchange agreement [20, 21]. We obtained dates of death from VHA vital status files. The institutional review boards of Vanderbilt University and the VHA Tennessee Valley Healthcare System approved this study with a waiver of informed consent.

### Study population, treatment initiation and intensification

The study population comprised veterans ≥18 years old who filled a prescription for a hypoglycemic medication after at least 180 days without any oral or injectable diabetic drug fill (new-users), and who received regular VHA care (VHA encounter or prescription fill at least twice in the past 365 days) [17]. Thus, veterans were required to have at least 365 days of available baseline data preceding their first eligible medication fill. The date of the qualifying first hypoglycemic medication fill was considered time zero (*t0*). We categorized patients based on the two most common initial treatments: metformin and sulfonylurea.

We followed all new-users of metformin from *t0* through the prescription of another agent. Treatment intensification, termed *t1*_*,*_ to the initial regimen was defined as receiving a prescription for a medication other than metformin. The observation period extended from *t1* through loss to follow-up; defined as the 181^st^ day of no contact with any VHA facility (inpatient, outpatient or pharmacy use), death or the end of the study (September 30, 2011). Cohort re-entry was not allowed, therefore, only the first medication fill which fulfilled inclusion criteria was included.

### HbA1c and BMI

Baseline HbA1c or BMI was defined as the value obtained closest to or at *t1*. We also collected HbA1c or weight values in each 30 day time-block after *t1* and if none was available then the last observed post-intensification value was carried forward for up to 24 months. BMI was calculated by dividing the weight in kilograms by the square of height in meters. The median of all available heights was used for the calculation of BMI. Implausible values were excluded. This included any HbA1c value <3 and >30 %; height <48 and >90 in.; and weight <50 and >700 lb.

### Covariates

Study covariates were collected from 24 months preceding *t1* and values closest to *t1* are reported (Additional file 1: Table S1). Covariates included: age, sex, race (white, black, other), fiscal year, indicators of healthcare utilization (hospitalization within last year, number of outpatient visits), physiologic variables (blood pressure, creatinine, HbA1c, low density lipoprotein levels, presence of proteinuria, and body mass index), duration of monotherapy before intensification of diabetes regimen (diabetes duration), selected medications, smoking, and presence of co-morbidities.

### Statistical analysis

We tracked diabetes medications on a day by day basis and evaluated when a prescription occurred for any hypoglycemic medication after *t1*. We described the proportion of metformin initiators who changed therapy at each time point after *t1* and the most common regimens. Patterns of medications use are shown from *t1* onward in stacked bar plots.

Using descriptive statistics, medians and interquartile ranges, we examined demographics of the population who intensified metformin monotherapy and those who did not intensify metformin therapy by 48 months after metformin initiation.

To explore changes in HbA1c and BMI, we plotted the monthly median values of HbA1c and BMI of intensified regimens with 95 % confidence intervals using the maximum Harrell-Davis standard error over time [22]. We evaluated HbA1c and BMI using two approaches for medication use classification. First, patients were considered to remain on their initial intensified regimen from treatment intensification (*t1*) through the end of their follow-up regardless of adherence to drug regimen, medication changes, or the development of contraindications (persistent exposure not required). Second, to minimize exposure misclassification, exposure was defined and follow-up began at *t1 + 6 months* and persistent exposure was required [23, 24]. Non-persistence was defined as a gap of 90 days in which the patient had no anti-diabetic therapy available. Non persistence to the initial or intensified regimen could also occur at the time the patient filled a third anti-diabetic drug. In our population, allowing 90 days to refill medications approximates adherence of 80 % [25]. Analyses were conducted using R Statistical Program (R Foundation, available at: http://www.r-project.org).

## Results

**Fig. 1:**
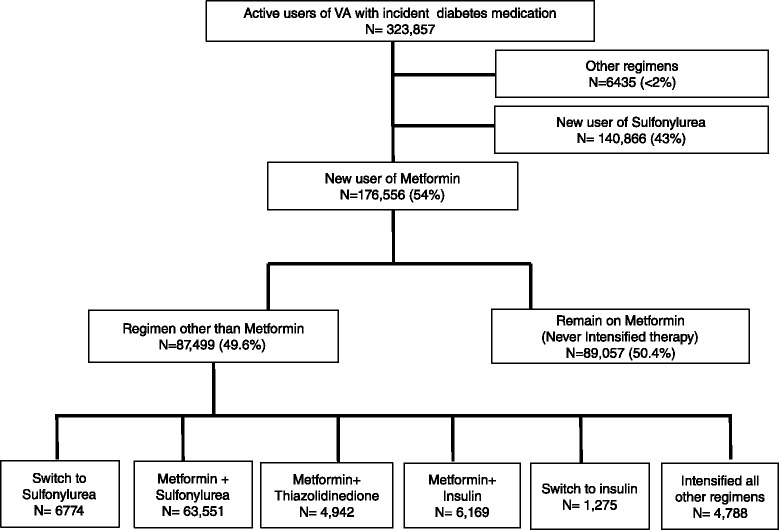
Cohort identification and flow

There were 323,857 new-users of hypoglycemic medications (Fig. 1). Of these, 176,556 (55 %) patients initiated metformin, 140,866 (43 %) initiated sulfonylurea, and 2 % initiated other regimens. Fifty percent (*N* = 89,057) of metformin initiators remained on metformin therapy over a median follow-up 58 months (interquartile range [IQR] 35, 74). There were 87,499 (49.6 %) patients who filled a prescription for a regimen other than metformin. The four most common intensification patterns were the addition of sulfonylurea (79 %), thiazolidinedione [TZD] (6 %), or insulin (8 %) or a switch to insulin monotherapy (2 %). The remaining 6 % included triple therapy, Glucagon like peptide-1 (GLP-1) receptor agonists and Dipeptidyl peptidase -4 inhibitors (DPP-4).

### Patterns of diabetes medication use over time

**Fig. 2:**
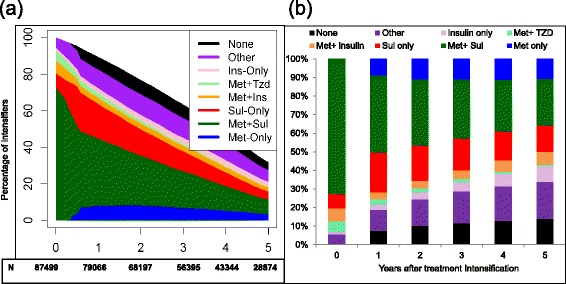
Diabetes drug intensification patterns among patients who initiated treatment for type 2 diabetes. The total proportion of initiators decreases over time because patients die or have less than 5 years of follow-up in cohort. Stacked bar plots represent drug patterns after fill of a medication other than metformin (panel **a**) Cross sectional proportion of diabetes drugs used among patients alive at each time-point (panel **b**)

Figure 2 panel a depicts the pattern of treatment regimens during 5 years post-intensification in the cohort of metformin initiators who intensified. All proportions are calculated based on the initial number of patients that intensified treatment (time 0 in figure). Thus, the figure also illustrates that the total number of metformin patients observed declined over time as patients died, were lost to follow-up or reached the end of study. Figure 2 panel b is a cross sectional evaluation of each year after intensification (where the proportion add to 100 %). A non-trivial proportion stopped all hypoglycemic medications (~10 % at any time point had no hypoglycemic filled within the prior 90 days). The use of sulfonylureas in combination or alone gradually decreased, but insulin use remained relatively low. For the 33 % of intensifiers who remained under follow-up at 5 years, 10.7 % were using metformin only, 25 % metformin + sulfonylurea, 14 % sulfonylurea only, 20 % other regimens, 15 % insulin based regimens and 14 % no treatment fill in the prior 90 days.

### Characteristics of intensifiers and non intensifiers

Patients who intensified metformin monotherapy were predominately white males with a median age of 62 years old. Median time on metformin monotherapy prior to intensification was 18 months (IQR 6, 37) for addition of sulfonylurea, 11 months (4,23) for addition of TZDs, 13 months (3,34) for addition of insulin, and 31 months (18, 49) for switching to insulin. The median follow-up after intensification was 47 months (26, 66). Patients who intensified their metformin with an oral agent (either sulfonylurea or TZD) had less co-morbidity, fewer outpatient visits and were less likely to have been hospitalized in the previous year than those who added insulin or switched to insulin (Table 2).

**Table 2:** Characteristics of patients who intensified metformin therapy

	*Metformin +Sulfonylurea*	*Metformin + TZD*	*Metformin +Insulin*	*Metformin switch to insulin*
N	63,551	4942	6169	1275
Age, years median IQR^a^	62 (56.5, 70.9)	65 (57.3, 73.9)	61 (54.7, 69.3)	66 (58.4, 76.7)
Male, (%)	96	95	95	96
Race, (%)				
White	74	78	68	74
Black	14	10	21	18
Other	4	6	5	5
Months to intensification^a^	18 (6, 37)	11 (4, 23)	13 (3, 34)	31 (18, 49)
Systolic Blood Pressure, mmHg^a^	132 (121, 142)	132 (120, 142)	130 (119, 142)	130 (119, 142)
Creatinine, mg/dL^a^	1.0 (0.9, 1.2)	1.0 (0.9, 1.2)	1.0 (0.9, 1.2)	1.1 (0.9, 1.3)
Hospitalized in the last year,%	17.8	17.7	42.9	50.6
Number of outpatient visits^a^	6 (4, 11)	5 (3, 9)	7 (4, 13)	6 (3, 12)
Comorbidities, %				
Malignancy	9	8	12	16
Liver/Respiratory disease	4	3	10	18
Congestive heart failure	8	6	13	24
Cardiovascular disease	32	33	38	52
Serious Mental illness	28	23	36	38
Arrhythmia	11	10	14	25
COPD/Asthma	17	14	24	32
Smoking	19	14	24	28
Medications				
ACE/ARB	71	69	67	52
Antihypertensives	74	73	73	67
Antiarrhythmics	2	2	4	4
Anti-coagulants	13	16	18	24
Statins/lipid lowering drugs	78	77	70	55
Nitrates	11	11	14	13
Aspirin	24	19	30	23
Loop diuretics	13	12	22	24

*IQR* interquartile range

^a^median and interquartile range reported

Characteristics of metformin patients who did not intensify therapy by 1, 2 and 4 years of follow-up are included in Additional file 2: Table S2. Patients who remained on metformin monotherapy (never intensified) at 4 years after metformin initiation were slightly older (median age 67.3 years [60.8, 75.8]) than the majority of patients who intensified treatment with metformin + sulfonylurea (62 years [56.5, 70.9]). The median HbA1c among those who remained on metformin monotherapy at 4 years was 6.4 % [6.0, 6.8], 46 mmol/mol [42, 51] and BMI was 30.3 (27.1, 34.3). The proportion with specific comorbidities was generally similar between those who remained on metformin monotherapy and those who intensified treatment.

### HbA1c and BMI

**Fig. 3:**
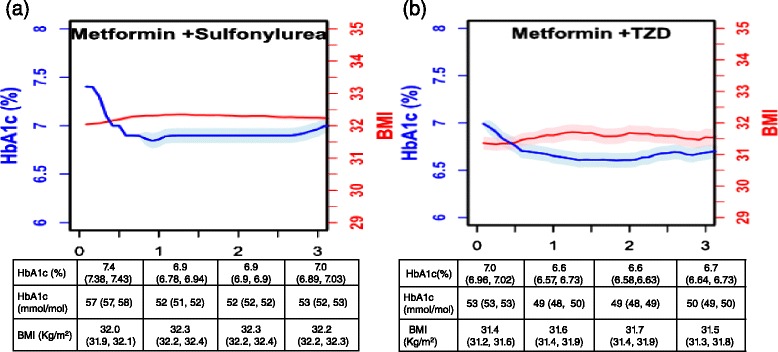
Median HbA1c and BMI and confidence intervals by intensification group over time: metformin+ sulfonylurea (Panel **a**); metformin+ thiazolidinedione (Panel **b**). Patients are allocated into these drug treatment exposures at treatment intensification persistence on medication is not required. Confidence intervals were calculated using the maximum Harrell-Davis standard error

**Fig. 4:**
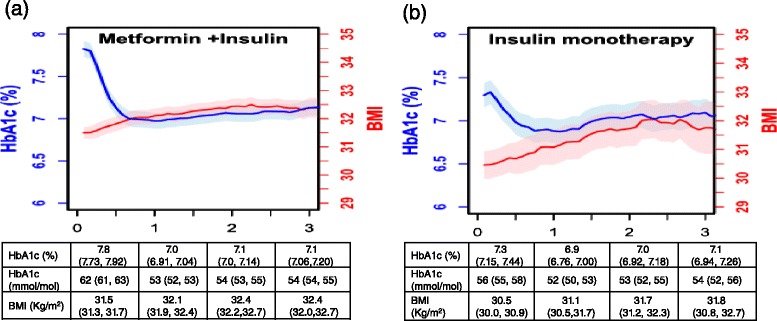
Median HbA1c and BMI and confidence intervals by intensification group over time: metformin+ insulin (Panel **a**); switch to insulin monotherapy (Panel **b**). Patients are allocated into these drug treatment exposures at treatment intensification persistence on medication is not required. Confidence intervals were calculated using the maximum Harrell-Davis standard error

We followed HbA1c and BMI of patients who intensified to four common regimens after metformin monotherapy through the end of study or death (persistent exposure not required). Across these regimens, median HbA1c values declined from a range of 7.0–7.8 % (53–62 mmol/mol) at intensification to 6.6–7.0 % (49–53 mmol/mol) at 1 year, and remained stable up to 3 years afterwards. Median BMI ranged between 30.5 and 32 kg/m2 at intensification and increased during follow-up very modestly in those who intensified with oral regimens (Fig. 3). This modest weight gain stabilized by 2 years after treatment intensification. Among those using insulin based regimens (Fig. 4), weight gain was more pronounced and yielded between 1.5 and 2 unit increase in BMI (approximately 3–4 kg weight gain for an average man 5 ft 10 in.). Weight gain among insulin regimens did not appear to stabilize by 3 years. A parallel analysis, which censored patients if they switch regimens (persistent exposure required), yielded similar results (Additional file 3: Figure S1).

## Discussion

In this study, we report two main findings. First, among a national cohort of veterans with diabetes, 50 % of metformin initiators intensified therapy by 4 years of follow-up and while sulfonylurea was the most common addition, there was an increase in the proportion adding insulin over time. Second, among those who intensified to the four most common regimens, the median HbA1c was between 7 and 7.8 % (53–62 mmol/mol) at intensification and 6.6 to 7 % (49–53 mmol/mol) one year later and remained about 7 % for the next 3 years. Those on insulin based regimens had a 1–2 kg/m2 weight gain over 3 years.

Our observation of reductions in HbA1c levels with an associated weight gain among insulin regimens is consistent with results reported by Liu and colleagues [26]. This network meta-analysis of 39 clinical trials (17,860 patients) compared addition of second line medication classes on HbA1c and BMI outcomes. Trials included were a minimum of 12 weeks and all less than a year of follow up, and evaluated metformin with the addition of either: placebo, sulfonylureas, TZDs, alpha-glucosidase inhibitors, glinides, DPP-4 inhibitors, or GLP-1 analogues or insulin. All drug classes reduced HbA1c by 0.6–1.07 % with no statistically significant differences among classes. Of note, changes in HbA1c were similar between regimens that added either insulin (biphasic insulin −1.07 % [−1.46, −0.69 %]; basal insulin −0.88 % [−1.21, −0.56 %]), or sulfonylureas (−0.82 % [−0.95, −0.70 %]) to metformin. However, biphasic insulin was noted to have the most weight gain (3.41 kg [2.04, 4.77]) followed by sulfonylureas (2.17 kg [1.70, 2.65 kg]) and basal insulin (1.38 kg [0.18, 2.60 kg]). In another meta-analysis by Bennett et al. [27] that included both clinical trials and observational studies, most add-on regimens had similar efficacy in reducing HbA1c but also differed in their effects on BMI.

In our cohort, the median HbA1c among those who intensified metformin (*N* = 87,499) was 7.4 % (6.7, 8.4) 57 mmol/mol (50, 68) at intensification, while the median HbA1c was 6.4 % (6.0, 6.8) 46 mmol/mol (42, 51) among those (*N* = 73,623) who did not intensify metformin monotherapy by 4 years. Interestingly, patients who intensified treatment were relatively similar in age and co-morbidities to those who did not intensify by 4 years. The American Geriatrics Society and the American Diabetes Association recommendations for care of the older adult with diabetes continue to recommend personalized HbA1c goals based on co-morbidities with appropriate goals being 7.5–8 % (58–64 mmol/mol) [28, 29]. VHA uses an approach which prompts the provider to risk stratify the patient to determine the HbA1c goals. Using this strategy many patients within VHA would fall under glycemic targets of <9 % (<75 mmol/mol) (Table 1) [13]. Further research is needed to determine which patients might benefit from more aggressive diabetes therapy rather than applying broad thresholds to all patients [30].

Some limitations to our study should be noted. First, insulin is available as an over the counter product which may result in underestimation and misclassification of insulin users. However, a previous validation of insulin filled through VHA pharmacies found high accuracy (positive predictive value = 88 % [80,93], negative predictive value = 95 % [92,97]), suggesting that over the counter insulin use is infrequent in our system [31]. Second, we utilized refill data as a proxy for medication taking. Nevertheless, prescription fills are a good proxy for medication use. Third, veterans may not receive all their care or medications in VHA facilities resulting in missing laboratory, weight data or medications, which we partially addressed through addition of Medicare and Medicaid information [20, 21]. Fourth, our patients reflect a typical veteran population, with most patients being white and male and with a more restricted formulary than many patients seen in the private sector. Extrapolation of these findings to other populations warrants caution. Finally, our assessment is purely descriptive and formal comparisons among intensification groups require accounting for the potential issues in confounding factors.

## Conclusion

In summary, we found that all regimens were associated with HbA1C declines at 1-year of follow-up, as expected from clinical trial results. Those prescribed insulin-based regimens were more likely to have a sustained increase in weight. Although this study was not designed to directly compare regimen effectiveness, given the difference in the treatment patterns, and the similarity in HbA1c reduction, more emphasis should be placed on identifying oral regimens which are acceptable to patients, affordable, and have minimal side effects such as weight gain or hypoglycemia. This finding of increased weight gain among those using insulin in combination with our previous finding of increased mortality risk [14] should be considered by patients and clinicians when discussing the risks and benefits of adding insulin versus a sulfonylurea. Similarly, the HbA1c level at which patients have their treatment intensified should be individualized to account for the co-morbidities, life expectancy and patient age.

## Additional files

Additional file 1: Table S1. Definitions of comorbid conditions and medications, on the basis of codes and prescriptions in 730 days (24 months) before treatment intensification. (DOC 79 kb) Additional file 2: Table S2. Characteristics of Metformin initiators who never intensified therapy by 12, 24 and 48 months of follow-up. (DOC 74 kb) Additional file 3: Figure S1. Median HbA1c and BMI and confidence intervals* by intensification group over time: metformin+ sulfonylurea (Panel A); metformin+ thiazolidinedione (Panel B); metformin+ insulin (Panel C); or switch to insulin monotherapy (Panel D). Patients are allocated into these drug treatment exposures at treatment intensification + 6 months and persistence on medication is required. Confidence intervals were calculated using the maximum Harrell-Davis standard error. (DOCX 328 kb)

## Abbreviations

BMI: body mass index
DPP-4 inhibitors: dipeptidyl peptidase -4 inhibitors
GLP-1 receptor agonists: glucagon like peptide-1 receptor agonists
HbA1C: glycated hemoglobin
IQR: interquartile range
*t0*: time zero—start of first diabetes drug
*t1*: time one- date of treatment intensification or additional medication added to first diabetes drug
TZD: thiazolidinedione
VHA: Veterans Health Administration
