# Supplementary material for: Diabetes treatment intensification and associated changes in HbA1c and body mass index: a cohort study
Source: BMC Endocr Disord. 2016 Jun 2;16:32. doi: 10.1186/s12902-016-0101-2 (PMC4890276; doi:10.1186/s12902-016-0101-2)
Supplement: Additional file 1: Table S1. — Definitions of comorbid conditions and medications, on the basis of codes and prescriptions in 730 days (24 months) before treatment intensification. (DOC 79 kb) [file 12902_2016_101_MOESM1_ESM.doc]

Supplemental Table 1: Definitions of comorbid conditions and medications, on the basis of codes and prescriptions in 730 days (24 months) before treatment intensification

| ***Covariate Condition*** | ***Inclusive conditions*** | ***Definition**** |
| --- | --- | --- |
| **Malignancy** | Cancer excluding non melanoma skin cancer | ICD 9- CM diagnosis codes:140.X-208.X (exclude 173) |
| **Liver/ Respiratory failure** | 1. End stage liver disease | ICD 9- CM diagnosis codes: 570.X- 573.X |
|  | 1. Respiratory failure | ICD 9- CM diagnosis codes: 518.81, 518.83, 518.84, 799.1, 415.X, 416.X |
| **Congestive Heart Failure** | CHF (excluding post procedure-CHF) | ICD 9- CM diagnosis codes: 428.X, 402.01, 402.11, 402.91, 404.01, 404.03, 404.11, 404.13, 404.91, 404.93, 425.X |
| **Cardiovascular disease** | 1. MI | ICD 9- CM diagnosis codes:410.X, 412.X, 429.7X |
|  | 1. Obstructive coronary disease | ICD 9- CM diagnosis codes:411.X, 413.X, 414.X  ICD9-CM procedure codes: 36.01, 36.02, 36.03, 36.05, 36.09, 36.10-36.19  CPT procedure codes: 33533-36, 33510-23, 33530, 92980-82,92984, 92995-6, 92974 |
|  | 1. TIA | ICD 9- CM diagnosis codes: 435.X |
|  | 1. Stroke | ICD 9- CM diagnosis codes: 430.X, 431.X. 434.X, 436.X |
|  | 1. Peripheral artery disease revascularization or amputation | ICD 9- CM diagnosis codes:440.2X, 442.2, 443.1, 443.9, 445.0X ICD9-CM procedure codes:38.08-09, 38.18, 38.38, 38.39, 38.48, 38.49, 38.88, 38.89, 39.25, 39.29, 39.5, 84.1X; 84.10-84.17  CPT procedure codes: 35226,35256, 35286, 35351, 35355, 35371, 35372, 35381, 35454, 35456, 35459, 35473, 35474, 35482, 35483, 35485, 35492, 35493, 35495, 35546, 35548, 35549, 35551, 35556, 35558, 35563, 35565, 35566, 35571, 35583, 35585, 35587, 35646, 35651, 35654, 35656, 35661, 35663, 35665, 35666, 35671, 34800, 34802-5 |
|  | 1. Carotid revascularization | ICD9-CM procedure codes: 38.12, 38.11, 00.61, 00.63, 39.28  CPT procedure codes: 35301, 0005T, 0006T, 0007T, 0075T, 0076T, 37215, 37216  HCPCS procedure code: S2211 |
|  | 1. Pentoxifylline & related drugs | Medications: Pentoxifylline, Cilostazol, Cyclandelate, Ethaverine HCL, Nicotinyl Alcohol Tartate, Papaverine, Tolazolin |
| **Serious Mental illness** | 1. Dementia | ICD 9- CM diagnosis codes: 290.X, 291.2, 292.82, 294.1X, 331.0-331.1X, 331.82  Medications: Donepezil, Rivastigmine, Galantamine, Tacrine, Memantine |
|  | 1. Depression, | ICD 9- CM diagnosis codes: 311, 300.4, 296.2, 296.3, V79.0 |
|  | 1. Schizophrenia, | ICD 9- CM diagnosis codes: 295.X |
|  | 1. Bipolar disorder | ICD 9- CM diagnosis codes: 296.0, 296.4X, 296.5X, 296.6X, 296.7, 296.80, 296.89 |
|  | 1. Post traumatic stress disorder | ICD 9- CM diagnosis codes: 309.81 |
| **Cardiac valve disease** |  | ICD 9- CM diagnosis codes: 394.X, 395.X, 396.X, 424.0, 424.1 |
| **Arrhythmia** | 1. Atrial fibrillation/flutter | ICD 9- CM diagnosis codes: 427.3X |
|  | 1. Arrhythmia and conduction disorder | ICD 9- CM diagnosis codes: 426.X, 427.X |
| **Smoking** |  | ICD 9- CM diagnosis codes:305.1, V15.82, 989.84  Medications: Varenicline tartrate, Nicotine Replacement therapy (gum, patch, lozenge) |
| **COPD/ Asthma** |  | ICD 9- CM diagnosis codes:491.X, 492.X, 493.X, 496.X, V17.5, V81.3 |
| **HIV** |  | ICD 9- CM diagnosis codes: 042, 079.53, 795.71, V08  Medications: Zidovudine, Didanosine, Zalcitabine, Stavudine, Indinavir, Ritonavir, Saquinavir, Nevirapine, Nelfinavir, Delavirdine, Delavirdine, Abacavir, Amprenavir, Efavirenz, Lamivudine-Zidovudine, Ritonavir-Lopinavir, Abacavir-Lamivudine-Zidovudine |
| **Parkinson’s Disease** |  | ICD 9- CM diagnosis codes: 332  Medications: Apokyn, Apomorphine, Carbidopa/levodopa, Entacapone, Pergolide, Pramipexole, Ropinirole, Rotigotine, selegiline, Tolcapone, Zelapar Azilect/rasagiline, Emsam, Isocarboxazid, Phenelzine, Tranylcypromine |
| **Medications** |  |  |
| **ACE Inhibitors or ARBs alone/combination** | ACE Inhibitors or ARBs alone/combination | Benazapril, Captopril, Enalapril, Fosinopril, Lisinopril, Moexipril, Perindopril, Quinapril, Ramipril, Trandolapril, Candesartan, Eprosartan, Irbesartan, Losartan, Azilsartan, Olmesartan, Telmisartan, Valsartan |
| **Antipsychotics** | Atypical and typical antipsychotic medications | Lithium, Clozapine, Haloperidol, Loxipine, Lurasidone, Molindone, Olanzipine, Paliperidone, Quetiapine fumerate; Resperidone, Aripiprazole, Asenapine, Ziprasidone, Chlorpromazine, Fluphenazine, Fluphenazine deconate, Mesoridazine, Perphenazine, Thioridazine, Thiothixene; Trifluoperazine; Triflupromazine, Asenapine, Chlorprothixene, Iloperidone, Molindone, Promazine, Piperacetazine, Methotrimeprazine, Acetophenazine |
| **Antihypertensives** | 1. Beta-blockers | Acebutolol, Atenolol, Betaxolol, Bisoprolol, Carteolol, Carvedilol, Esmolol, Labetalol, Metoprolol Tartrate, Metoprolol Succinate, Propranolol, Penbutolol, Pindolol, Nadolol, Sotolol, Timolol, Nebivolol |
|  | 1. Calcium Channel Blockers | Amlodipine, Isradipine; Felodipine, Nifedipine, Nifedipine ER, Nicardipine; Diltiazem, Verapamil, Nimodipine; Nisoldipine; Bepridil, Amlodipine–Atorvastatin, Clevidipine Butyrate, |
|  | 1. Thiazide diuretics/ Potassium sparing diuretics | Chlorothiazide, Chlorthalidone, Hydrochlorothiazide, Methyclothiazide, Trichlormethiazide, Metolazone, Indapamide, Eplerenone; Ameloride, Sprinolactone, Triamterene, Hydrochlorothiazide/Triamterene, Hydrochlorothiazide/Spironolactone, Bendroflumethiazide, Benzthiazide, Cyclothiazide, Hydroflumethiazide, Methyclothiazide, Trichlormethiazide, Metolazone, Indapamide, Polythiazide, Quinethazone |
|  | 1. Other Antihypertensives | Doxazosin, Prazosin, Terazosin, Clonidine, Guanabenz, Guanfacine, Hydralazine, Methyldopa, Metyrosine, Reserpine, Minoxidil, Alfuzosin, Silodosin, Alseroxylon, Cryptenamine, Deserpidine, Diazoxide Guanethidine, Iloprost, Mecamylamine, Pargyline, Rescinnamine, Trimethaphan Camsylate |
| **Anti-arrhythmics Digoxin and other inotropes** | 1. Digoxin | Digoxin, Digitalis |
|  | 1. Anti- Arrythmics | Adenosine, Amiodarone, Lidocaine, Flecainide, Ibutilide, Pacerone, Procainamide, Rhythmol, Propafenone, Quinidine, Disopyramide, Verapamil, Dofetilide, Mexiletine, Moricizine, Tocainide |
| **Anticoagulants and Platelet inhibitors, not aspirin** | 1. Anticoagulants | Warfarin, Argatroban, Bivalirudin, Dalteparin, Enoxaprin, Eptifibatide, Fondaparinux, Heparin, Lepirudin, Tirofiban, Tinzaparin, Reviparin, Nadroparin, Ardeparin, Certoparin, Dabigatran |
|  | 1. Platelet Inhibitors | Clopidogrel, Ticlopidine, Aspirin/ Dipyrimidole, Dipyrimidole alone, Abciximab, Factor IX, Factor VIIa, Factor VIII, Prasugrel, Ticagrelor |
| **Lipid lowering drugs** | 1. Statins | Atorvastatin, Fluvastatin, Lovastatin, Pravastatin, Simvastatin, Rosuvastatin, Cerivastatin Pitavastatin, Lovastatin ER, Ezetamibe/Simvastatin, Lovastatin /Niacin |
|  | 1. Non Statins | Cholestyramine, Colesevelam, Clofibrate, Colestipol, Niacin, Niacinamide, Fish Oil Concentrate, Omega 3 Fatty Acids, Gemfibrozil, Fenofibrate, Fenofibric Acid, Ezetimibe Omacor, Tricor/Fenofibrate, Ezetamibe/Simvastatin |
| **Nitrates** |  | Amyl nitrate, Isosorbide Dinitrate, Isosorbide mononitrate, Erythrityl Tetranitrate Nitroglycerin (all forms--SA, Patch, SL, Ointment; Aerosol spray), Ranolazine |
| **Aspirin** |  | Aspirin, Aspirin/ Dipyrimidole |
| **Loop Diuretics** |  | Furosemide, Ethacrynic acid, Bumetanide, Torsemide |
