# Supplementary material for: Diabetes treatment intensification and associated changes in HbA1c and body mass index: a cohort study
Source: BMC Endocr Disord. 2016 Jun 2;16:32. doi: 10.1186/s12902-016-0101-2 (PMC4890276; doi:10.1186/s12902-016-0101-2)
Supplement: Additional file 2: Table S2. — Characteristics of Metformin initiators who never intensified therapy by 12, 24 and 48 months of follow-up. (DOC 74 kb) [file 12902_2016_101_MOESM2_ESM.doc]

Supplemental Table 2: Characteristics of metformin initiators who never intensified therapy by certain follow-up time points.

|  | ***No intensification by 12 months*** | ***No intensification by 24 months*** | ***No intensification by 48 months*** |
| --- | --- | --- | --- |
| **N** | 135,005 | 108,152 | 73,623 |
| **Age**, years median IQR | 63.5 ( 57.1, 72.7) | 64.9 (58.4, 73.9) | 67.3 (60.8, 75.8) |
| **Male,** (%) | 95 | 95 | 95 |
| **Race,** (%) |  |  |  |
| White | 76 | 77 | 77 |
| Black | 13 | 12 | 12 |
| Other | 4 | 4 | 4 |
| Available,% | 93 | 93 | 93 |
| **Body mass index** (Kg/m^2^), median (IQR) | 31.0 (27.6, 35.1) | 30.7 (27.4, 34.8) | 30.3 (27.1, 34.3) |
| Available,% | 92.9 | 98.1 | 97.8 |
| **HbA1c** (%)**,** median (IQR) | 6.4 (6.0, 6.9) | 6.4 (6.0, 6.8) | 6.4 (6.0, 6.8) |
| Available,% | 76.7 | 90.6 | 91.7 |
| **Systolic Blood Pressure,** mmHg median (IQR) | 130 (120, 140) | 130 (120, 139) | 129 (120, 138) |
| **Diastolic Blood Pressure,** mmHg median (IQR) | 74 (68, 81) | 74 (67, 81) | 73 (66, 80) |
| Available,% | 94.3 | 98.8 | 98.5 |
| **LDL cholesterol,** mg/dL median (IQR) | 92 (74, 114) | 88.4 (72, 109) | 84 (68, 103) |
| Available,% | 69.1 | 87.2 | 91.0. |
| **Creatinine,** mg/dL median (IQR) | 1.0 (0.9, 1.2) | 1.0 (0.9, 1.19) | 1.0 (0.9, 1.16) |
| **Creatinine,** μmol/L median (IQR) | 88.4 (79.6, 106.1) | 88.4(79.6,105.2) | 88.4(79.6,102.5) |
| Available | 79.2 | 90.7 | 89.9 |
| **Urine Protein,** negative% |  |  |  |
| Trace to 1+ | 15.8 | 16.9 | 19.6 |
| 2+ to 4+ | 2.8 | 2.8 | 3.0 |
| Available,% | 66.0 | 62.2 |  |
| **Hospitalized in the last year,**% | 16.6 | 15.4 | 13.2 |
| **Number of outpatient visits,** median (IQR) | 6 (3,10) | 5 (3,9) | 5 (2,9) |
| **Comorbidities**, % |  |  |  |
| Malignancy | 8.1 | 9.8 | 12.5 |
| Liver/ Respiratory disease | 2.8 | 3.6 | 4.9 |
| Congestive heart failure | 5.6 | 6.7 | 8.3 |
| Cardiovascular disease | 29.6 | 33.8 | 38.5 |
| Serious Mental illness | 23.2 | 26.9 | 31.9 |
| Cardiac valve disease | 2.0 | 2.7 | 3.8 |
| Arrhythmia | 9.2 | 11.6 | 15.0 |
| COPD/Asthma | 15.0 | 17.6 | 20.0 |
| Smoking | 15.6 | 18.5 | 22.1 |
| HIV | 0.3 | 0.3 | 0.3 |
| **Medications** |  |  |  |
| ACE /ARB | 60.2 | 62.9 | 62.5 |
| Antipsychotics | 7.0 | 7.2 | 7.3 |
| Antihypertensives | 68.5 | 70.2 | 71.1 |
| Antiarrhythmics | 1.4 | 1.6 | 1.9 |
| Anti-coagulants | 11.9 | 12.6 | 12.7 |
| Statins and other lipid lowering drugs | 69.6 | 73.3 | 75.6 |
| Nitrates | 9.0 | 8.9 | 7.6 |
| Aspirin | 18.5 | 19.5 | 20.1 |
| Loop diuretics | 9.6 | 9.7 | 9.7 |

ACEI = angiotensin-converting enzyme inhibitor; ARB = angiotensin-receptor blocker; COPD = chronic obstructive pulmonary disease; CPT = Current Procedural Terminology; ICD-9- CM = International Classification of Diseases, Ninth Revision; MI = myocardial infarction; TIA = transient ischemic attack

* Each co-morbid condition was defined as present if there was 1 specified inpatient or 2 specified outpatient codes separated by 30 days, or 1 specified procedure code or prescription for a medication defining that comorbid condition in the 730 days before treatment intensification.
