# Supplementary figures and images for: Diabetes treatment intensification and associated changes in HbA1c and body mass index: a cohort study
Source: BMC Endocr Disord. 2016 Jun 2;16:32. doi: 10.1186/s12902-016-0101-2 (PMC4890276; doi:10.1186/s12902-016-0101-2)

**
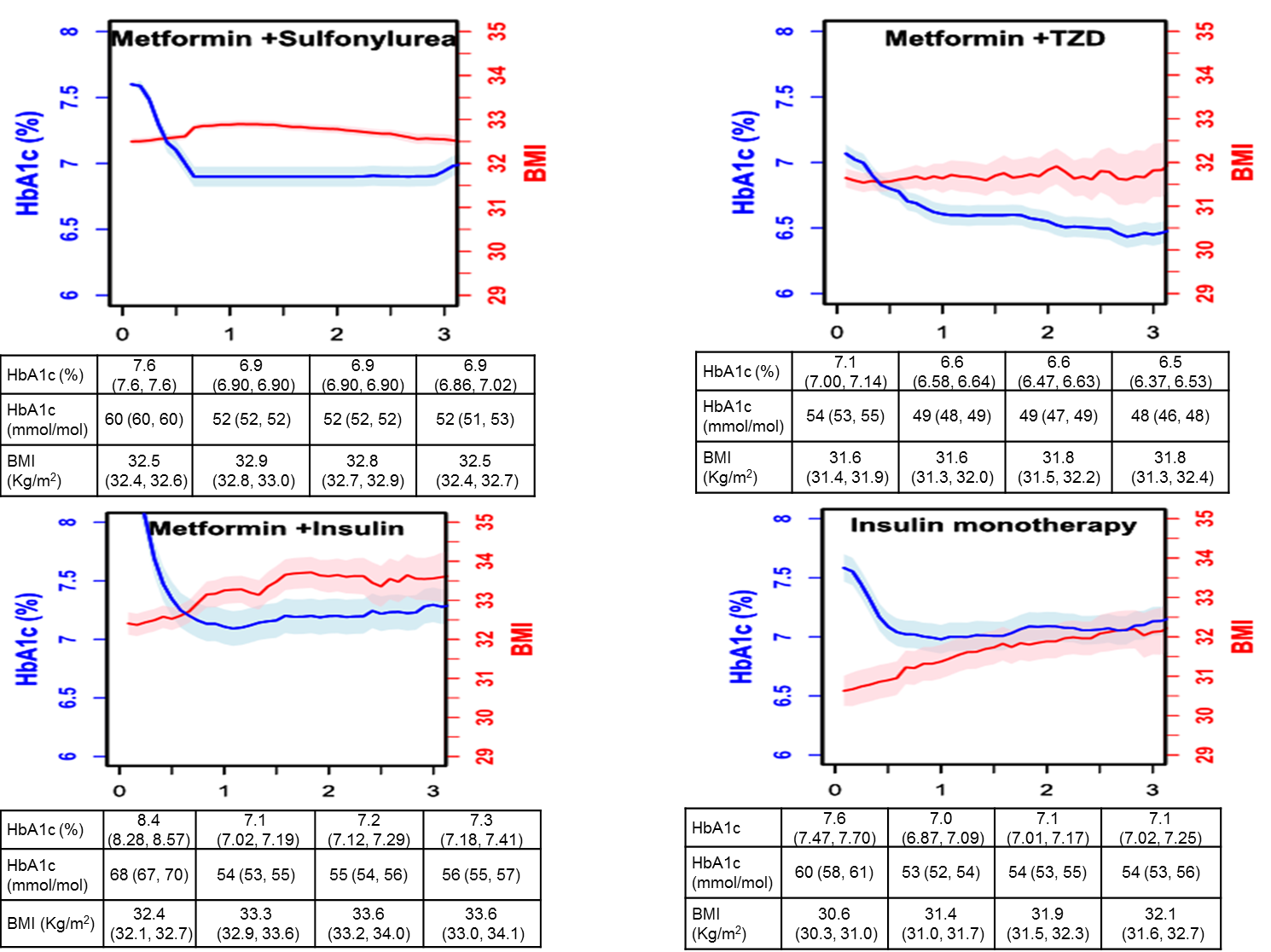
**

Supplement: Additional file 3: Figure S1. — Median HbA1c and BMI and confidence intervals* by intensification group over time: metformin+ sulfonylurea (Panel A); metformin+ thiazolidinedione (Panel B); metformin+ insulin (Panel C); or switch to insulin monotherapy (Panel D). Patients are allocated into these drug treatment exposures at treatment intensification + 6 months and persistence on medication is required. Confidence intervals were calculated using the maximum Harrell-Davis standard error. (DOCX 328 kb) [file 12902_2016_101_MOESM3_ESM.docx]
